# Lateral-flow assays for bovine paratuberculosis diagnosis

**DOI:** 10.3389/fvets.2023.1257488

**Published:** 2023-10-12

**Authors:** Marta Alonso-Hearn, Ana Ballesteros, Alejandra Navarro, Gerard Badia-Bringué, Rosa Casais

**Affiliations:** ^1^Department of Animal Health, NEIKER-Basque Institute for Agricultural Research and Development, Basque Research and Technology Alliance, Derio, Bizkaia, Spain; ^2^Biolan Health S.L, Technological Park of Bizkaia, Zamudio, Bizkaia, Spain; ^3^SERIDA, Servicio Regional de Investigación y Desarrollo Agroalimentario, Center of Animal Biotechnology, Deva, Asturias, Spain

**Keywords:** Paratuberculosis, lateral flow assays, biosensors, *Mycobacterium avium* subsp. paratuberculosis, cattle, biomarkers

## Abstract

*Mycobacterium avium* subsp. *paratuberculosis* (MAP) causes bovine paratuberculosis (PTB). PTB is responsible for significant economic losses in dairy herds around the word. PTB control programs that rely on testing and culling of test-positive cows have been developed. Current diagnostics, such as ELISA for detecting MAP antibodies in serum samples and PCR detecting MAP DNA in feces, have inadequate sensitivity for detecting subclinical animals. Innovative “omics” technologies such as next-generation sequencing (NGS) technology-based RNA-sequencing (RNA-Seq), proteomics and metabolomics can be used to find host biomarkers. The discovered biomarkers (RNA, microRNAs, proteins, metabolites) can then be used to develop new and more sensitive approaches for PTB diagnosis. Traditional approaches for measuring host antibodies and biomarkers, such as ELISAs, northern blotting, quantitative reverse-transcriptase polymerase chain reaction (RT-qPCR), cDNA microarrays, and mass spectrometry are time-consuming, expensive, and sometimes exhibit poor sensitivity. With the rapid development of nanotechnology, low-cost monitoring devices for measuring antibodies against MAP proteins in point-of-care (POC) settings have been developed. Lateral flow assays (LFAs), in particular, are thought to be appropriate for the on-site detection of antibodies to MAP antigens and/or host biomarkers. This review aims to summarize LFAs that have recently been developed to accurately detect antibodies against MAP antigens, as well as the benefits that host biomarkers linked with MAP infection give to PTB diagnosis. The identification of these novel biomarkers could be the basis for the development of new LFAs. The dairy industry and producers are likely to benefit from reliable and rapid technologies capable of detecting MAP infection *in situ* to establish a quick and sensitive PTB diagnosis.

## 1. The economic and social consequences of bovine paratuberculosis (PTB)

Infection caused by *Mycobacterium avium* subsp. *paratuberculosis* (MAP) in domestic and wild ruminants is recognized as a global major issue in animal health by the World Organization for Animal Health (WOAH), which requires member countries to maintain epidemiological surveillance and notify disease cases. In cattle, MAP infection induces a chronic wasting disease characterized by diarrhea and progressive loss of body condition (1). Due to lower milk production, increased management expenses, and premature culling or death from clinical disease, PTB is responsible for significant losses in dairy herds worldwide (2–4). More than half of dairy cattle herds in the United Stated and Europe test positive for MAP antibodies, indicating that bovine PTB is endemic in these areas (5–7). The economic impact of PTB on the US dairy sector has been estimated to range between $250 million and $1.5 billion per year, with a net return of over $100 per cow lower in a positive herd than in a negative herd (8). The annual economic impact of PTB in Europe has been estimated to be 364.31 million dollars (9). Furthermore, MAP is resistant to pasteurization and may enter the human food chain via meat, dairy products, and untreated water supplies (10). MAP is thought to be a cause of Crohn’s disease (CD) in humans and has been found in samples from patients with CD, ulcerative colitis, and idiopathic inflammatory bowel disease (IBD)-associated colorectal cancer (11–14). MAP has been proposed as a potential trigger factor in several human autoimmune disorders, including rheumatoid arthritis, multiple sclerosis, and type I diabetes (15–18).

## 2. Influencing factors on PTB control

Commercially available inactivated vaccines against bovine PTB are particularly effective in reducing MAP presence in feces and tissues, as well as increasing milk production and cow productive life in infected farms (19, 20). On the other hand, PTB immunization using heat-inactivated vaccines is prohibited in most European nations due to interference with *Mycobacterium bovis* detection tests (21). Currently, PTB control is based on testing and culling all test-positive cows, as well as minimizing MAP transmission to susceptible animals by improving on-farm biosecurity measures (22, 23). Lack of compliance with management guidelines, the use of tests with limited sensitivities to detect all the infected cattle, and the purchase of infected replacement animals contribute to the failure of such control programs (24). Infection occurs in the early months of life, largely through the fecal-oral route, but clinical onset occurs only around calving, when animals are 18 months or older. PTB-associated histological lesions were classified as focal, multifocal, and diffuse (diffuse paucibacillary or lymphoplasmacytic, diffuse intermediate, or diffuse multibacillary or histiocytic) (25, 26). The sensitivity of diagnostic tests significantly influences the success of “test and cull” control programs. Current diagnostic methods include enzyme-linked immunosorbent assays (ELISA), which detect antibodies against MAP in serum samples, and polymerase chain reaction (PCR), which detects MAP DNA in feces (Table 1). Currently, fecal culture is considered “the gold standard” technique for MAP infection diagnosis (27). Individual fecal culture, on the other hand, is expensive, time-consuming (from 5 weeks to 6 months for colonies to grow in solid media), and typically detects advanced forms of the disease because to the late onset of fecal shedding during the natural course of MAP infection. The sensitivity of the fecal culture in clinical animals is 70%, but it is only 23–29% in subclinical animals (28). ELISA, which detects serum and milk antibodies against MAP in infected animals, is routinely used to detect MAP infection. Although serum ELISA is a straightforward, quick, and inexpensive method of diagnosis, it has a low sensitivity for detecting subclinical animals. Serum ELISA sensitivity varies from 50 to 87% in cattle with clinical signs to 24–94% in cattle with no clinical signs but shedding MAP and 7–22% in infected cattle with no clinical signs and no shedding (28). The interferon-gamma (IFNγ) release assay (IGRA) detects host cell-mediated immune responses in early-stage MAP infections, but it needs to be improved because the purified protein derivative (PPD) antigen preparations used in the whole-blood stimulation cross-react with antigens from other environmental mycobacterial species (29). It is obvious that detecting subclinical infections remains difficult, and novel approaches are required to detect MAP-infected animals to control disease spread. New host biomarkers are needed to improve the next generation of PTB detection technologies.

**Table 1:** Summary of current and emerging MAP diagnostic tests.

	Name of test (“omic”)	Direct	Indirect	Stage of the infection
Current test				
	MAP Bacteriological Culture	x		Clinical
	PCR for the detection of MAP DNA	x		Clinical
	ELISA for the detection of antibodies against MAP		x	Clinical
	IFNγ release assay		x	Subclinical
Emerging test				
	Droplet digital PCR	x		Unknown
	Biomarker-based RT-qPCR (mRNA-Seq)		x	Unknown
	Biomarker-based ELISA (Proteomics)		x	Unknown
	Biomarker-based RT-qPCR (miRNA-Seq)		x	Unknown
	Biomarker-based MS (Metabolomics)		x	Unknown

## 3. Innovative -omics- technologies used to identify novel biomarkers

Next,-generation sequencing (NGS) technology-based RNA-sequencing (RNA-Seq), proteomics and metabolomics have immense potential since they enable the study of PTB pathogenesis and can be used to discover biomarkers for the development of innovative diagnostic tools, drugs, or vaccines (30). Transcriptomic, proteomic and metabolomic profiling of blood samples yields biomarkers for MAP infection (mRNAs, microRNAs, host proteins and metabolites) that can represent the entire spectrum of the disease, from the earliest signs to the most advanced stages (Table 1). A good biomarker for PTB diagnosis should be able to distinguish between infected and non-infected animals with high sensitivity and specificity. Although several MAP infection-associated host biomarkers have been found using transcriptomic and proteomic analysis (31–34), they have not been fully validated in naturally infected cattle at various stages of infection. In a recent work, RNA-Seq was used to identify host genes that were differentially expressed in peripheral blood (PB) samples collected from animals with distinct PTB-associated lesions (focal and diffuse) versus control animals with no lesions in gut tissues (35). This RNA-Seq analysis enabled the identification of a candidate bovine biomarker, the ATP-binding cassette sub-family A member 13 (ABCA13), which was found to be overexpressed in the PB of MAP-infected Holstein cows with focal lesions versus control animals with no lesions in gut tissues. Recently, an ELISA designed to detect the ABCA13 showed good discriminatory power between infected animals with focal lesions and non-infected animals, thus improving the diagnostic performance of the IDEXX ELISA and other traditional diagnostic methods (36). These results were corroborated by using a larger set of well-characterized plasma samples from MAP-infected cows (*N* = 566) and negative controls (*N* = 138) (37). Importantly, bovine ABCA13 was detected in the absence of prior bovine purified protein derivative (PPD) stimulation.

The differences in host protein concentrations/levels in blood and feces in response to MAP infection may be of diagnostic value. Various proteomic platforms have been explored. Using iTRAQ, a liquid chromatography and tandem mass spectrometry (LC–MS) approach, Seth et al. found transthyretin, retinol-binding proteins, and cathelicidin in serum samples from MAP-infected animals (30). You et al. identified six proteins up-regulated at least 2-fold in MAP-positive cows using 2D-dimensional Fluorescence Difference Gel Electrophoresis (2D-DIGE), including transferrin, actin-binding protein, complement subcomponent C1r, complement component C3, amine oxidase-copper containing 3 (AOC3), and thrombin (31). Espinosa et al. measured serum levels of haptoglobin and serum amyloid A, two inflammatory acute-phase proteins, in 190 naturally infected animals classified according to the pathological forms of infection (59 uninfected animals with no lesions, 73 with focal lesions, 19 with multifocal, 11 with diffuse paucibacillary, and 28 with diffuse multibacillary lesions) (38). Their findings revealed a significant increase in the levels of these proteins in the serum of the infected animals with focal lesions, low bacterial load, and with predominance of cell-mediated immune responses. The authors concluded that these proteins could be useful as early infection biomarkers, particularly for identifying subclinical animals. Park et al. identified the alpha-2-macroglobulin (A2M) as a new promising biomarker for enhancing MAP detection (39). They discovered that serum A2M levels were significantly higher in subclinical shedders (*N* = 27), subclinical non-shedder (*N* = 50), and clinical shedders (*N* = 18) than in a healthy control group (*N* = 11) from a PTB-free farm. Even though the study included a reduced number of healthy control cows due to the difficulty in finding a PTB-free farm, A2M ELISA demonstrated superior diagnostic performance (90.4% sensitivity and 100% specificity) than two commercial ELISAs for the detection of anti-MAP antibodies.

Biomarkers include host non-coding RNAs (ncRNAs) as well as mRNAs, proteins or metabolites. MicroRNAs are one of the most studied types of ncRNAs. MicroRNAs (18–25 nucleotides long) are highly conserved small RNAs that primarily regulate gene expression by lowering the stability of their target mRNAs (40). Because microRNA-mRNA binding results in either mRNA cleavage or translational suppression, microRNAs are important regulators of gene function. Because of their significance in various diseases and their stability in biofluids, microRNAs have emerged as promising candidates with a vast diagnostic potential. Despite several freeze–thaw cycles or extreme pH, microRNAs exhibit remarkable stability (41). Further, microRNAs are measured with a high sensitivity and are suitable biomarker candidates in diagnosis compared to protein biomarkers, which are easily degraded over time. The identification of differentially expressed microRNAs between infected and not infected cattle suggests that microRNAs could be useful diagnostic biomarkers of MAP infection (34, 42–44).

Metabolomics measures chemical phenotypes that are the result of the activity on the transcriptome and proteome levels. Metabolomics has emerged as a method for characterizing the metabolic profiles of MAP-infected cattle. Sera from calves infected at 2 weeks of age were analyzed by 1H nuclear magnetic resonance spectrometry and compared to aged-matched controls in a monthly follow-up for 17 months (45). The same distinctive profile was detected at 3 months and 12 months after infection. Changes in acetone, citrate, glycerol and iso-butyrate concentrations suggested energy shortages and increased fat metabolism in infected cattle, whereas changes in urea and several amino acids indicated increased protein turnover. In a prospective study, cohorts of heifers and cows (*N* = 356) were followed up annually for 2–4 years using direct analysis in real time coupled with high resolution mass spectrometry (DART-HRMS) (46). Infected animals had increased levels of tryptamine and creatine/creatinine, but decreased levels of urea, glutamic acid and/or pyroglutamic acid. However, until 200 days post-infection, the metabolites identified by de Buck et al. (45) exhibited similar levels between MAP-infected and control cattle. Similarly, Tata et al. (46). discovered metabolites with overlapping levels between infected, infectious and control groups. To examine the time dependent changes following MAP infection in youngstock, Holstein Friesian calves were experimentally inoculated with MAP and along with 20 control calves, were sampled biweekly up to 13-months of age and subsequently monthly up to 19-months of age (47). Sera were assessed using flow infusion electrospray high-resolution mass spectrometry (FIE-HRMS) and out of 33 identified metabolites, six fatty acyls were able to distinguish between experimental groups throughout the study, including 8, 11, 14-eicosatrienoic acid and cis-8, 11, 14, 17-eicosatetraenoic acid. Furthermore, several metabolites that were suggested to be biomarkers for MAP in naturally infected heifer calves (48) were shown to be elevated in this study where heifer calves were experimentally inoculated.

## 4. Point-of-care (POC) platforms

Current PTB diagnostics require transporting the biological sample from the farm to the laboratory, as well as highly trained laboratory personnel and multistep preparation. As a result, the interval between sampling and outcome is long. Because of the rapid development of nanotechnology, it is now possible to develop quick and inexpensive monitoring devices that can aid in the measurement of host antibodies, microRNAs, and proteins in POC settings or resource-limited facilities (49, 50). The World Health Organization (WHO) has established the ASSURED criteria for an ideal POC assay, which stands for: affordable, sensitive (low number of false negatives), specific (low number of false positives), user-friendly (easy to perform), rapid and robust, equipment-free, and deliverable to those in need (51).

Nanoparticles (NPs) offer unique qualities such as high surface area to volume ratio, high surface energy, tunable absorption and emission properties, high stability, and biocompatibility, making them ideal for the designing of POC platforms (52). Because of their outstanding features that result in signal amplification, gold nanoparticles (AuNPs) have been widely used in biosensing. Their surface plasmon resonance (SPR) properties, in particular, make them excellent enhancers of the SPR electromagnetic field, resulting in increased signal amplification and sensitivity. SPR occurs in the visible range of the spectrum in AuNPs and is responsible for their outstanding optical features, such as size/aggregation-dependent color changes and high extinction coefficients. The aggregation of AuNPs results in interparticle surface plasmon coupling, which shifts the characteristic surface plasmon band (~ 520 nm) to longer wavelengths (~ 650 nm). As result, when dispersed in a solution, AuNPs appear reddish, changing to bluish when aggregated. Because such phenomenon occurs in the visible range of the spectrum, these changes can be detected with a standard ultraviolet–visible (UV–Vis) spectrophotometer or even the naked eye, eliminating the need for additional expensive instrumentation, making it suited for POC diagnostics.

Because of their innate biocompatibility, low cytotoxicity, and high stability in biological fluids, AuNPs are excellent materials for detecting host antibodies, microRNAs, and proteins in biological samples. AuNPs can be easily functionalized with biomolecules that have high affinity for these analytes. Adsorption-based, covalently biding-based, and affinity-based approaches are the most often used methods for functionalizing the surface of AuNPs. The adsorption-based method is based on electrostatic or hydrophobic interactions between the ligand and the surface of the AuNPs. The ligand is adsorbed onto the surface of the AuNPs, forming a non-covalent bond. In the covalent binding-based method, the ligand is covalently linked onto the surface of the AuNP using a thiol group. This can be accomplished by directly conjugating a sulfur-containing molecule or using a bi-functional linker. The linker has a thiol group at one end that has a high affinity for the AuNP surface, resulting in a semi-covalent bond (S-Au), and another functional group at the other end that allows other biomolecules to be attached. The affinity-based surface modification method involves functionalizing the AuNP surface with moieties that provide specific binding sites for biomolecule coupling. These affinity sites allow the selective attachment of biomolecules to the AuNP surface.

In the case of microRNAs, AuNPs are often functionalized with oligonucleotides containing a domain complementary to the microRNA’s target sequence. In the case of protein detection, AuNPs are functionalized with specific antibodies against the target protein. AuNP-based biosensors are categorized into two types: solution-based biosensors and solid support-based biosensors. The first describes biosensing devices that use AuNPs dispersed in solution (for example, colorimetric and fluorescent sensors). In the latter group, AuNPs are attached to a solid support (e.g., electrochemical sensors, SPR sensors, glass slide-based sensors, lateral-flow strip assays).

## 5. Lateral flow assays

**Figure 1:**
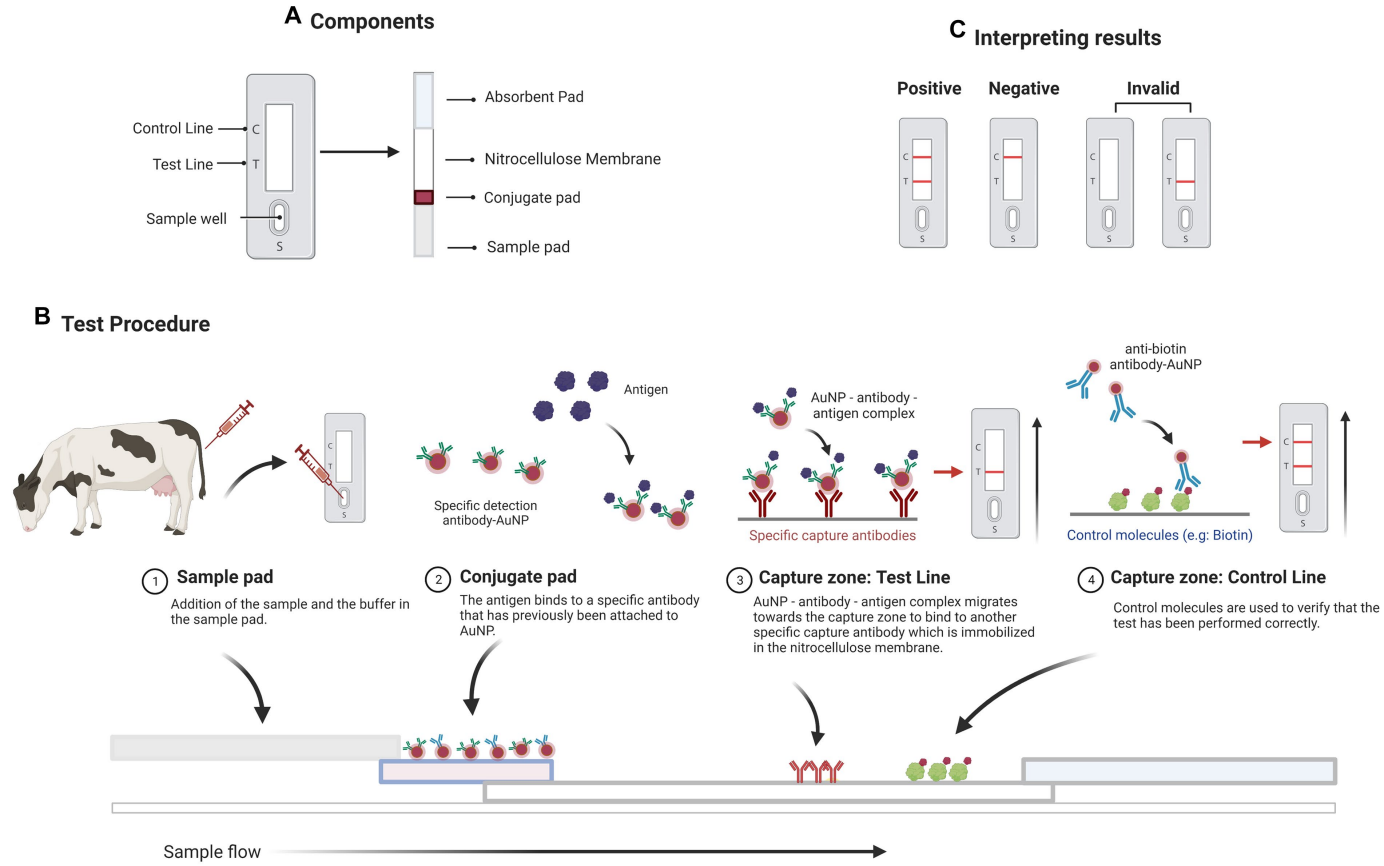
A sandwich lateral flow immunoassay (LFI). **(A)** The test strip contains several components including a sample pad, a detector conjugate pad, an absorption pad, and one nitrocellulose membrane with test and control lines. **(B)** Schematic representation of the operation of a LFI for detecting a specific antigen. **(C)** The appearance of two bands indicates a positive test result. The presence of only the test line or no line indicates that the test is invalid. The presence of simply the control line indicates a negative result. Created with BioRender.com.

Lateral flow assays (LFAs) are diagnostic assays based on the principles of immunochromatographic lateral flow test strips. The test sample flows along a solid substrate through capillary action, which takes 10–15 min to complete. LFAs are quick assays, which only require a few drops of a sample diluted in a buffer. LFAs are made up of a membrane (frequently made of nitrocellulose), a sample pad (made of cellulose or glass fiber), a conjugate pad (made of glass fiber), and an absorbent or wicking pad (typically a cellulose filter), all of which are laminated into a sheet of plastic known as the backing card (Figure 1A).

When a test sample containing the target analyte (for instance a protein) is added to the sample pad, it flows through the membrane solubilizing the conjugate (an AuNP-antibody) that flows alongside it (Figure 1B). The conjugate binds to the target present in the test sample, and the target-conjugate complex formed is captured at the test line by the capture elements (a secondary antibody) immobilized at this zone. In the test line, a colorimetric signal arises that is proportional to the amount of target present in the sample. The excess of the sample reaches a control line with a second conjugate (known as the control conjugate), confirming the proper flow of the liquid through the strip. Finally, an absorbent pad wicks the liquid away at the strip’s end. Aside from observing the test and control bands, their optical intensities can be determined using a portable strip reader and appropriate software (Figure 1C). LFAs are excellent tools for the on-site detection of host antibodies, proteins, or microRNAs in biological samples due to their intrinsic advantages, such as ease of use, low cost, and fast response (53). They can be classified into two categories based on the target analyte: Lateral Flow Immunoassays (LFIs, when the target is an antigen or an antibody) and Nucleic Acid Hybridization-based Lateral Flow Assays (NALFAs, when the target is a DNA or RNA sequence). LFIs are commonly used in the sandwich and the competitive formats (53). In the sandwich format, the conjugate binds to the target antigen present in the sample and then, the sample excess interacts with a control conjugate and forms a complex on the control line (54). In the competitive format, the antigen in the test sample competes with the immobilized antigen at the test zone for binding to the antibody conjugated with AuNPs.

In the case of microRNA detection, NALFAs are typically sandwich assays that rely on the complementary binding between the target microRNA sequence in the sample and both membrane-immobilized and AuNP-labeled oligonucleotide probes. The probes are immobilized in the membrane by high-affinity interactions between protein pairs, such as biotin-avidin/streptavidin. Typically, two biotinylated DNA probes, the capture and the control probes, are conjugated with streptavidin and distributed on the nitrocellulose membrane, forming the test and control lines, respectively. A detection probe complementary to the 3′ half of the target microRNA is conjugated to AuNPs via its thiol group, and this DNA-AuNPs conjugate is loaded on the conjugate pad. When the microRNA-containing sample is applied to the sample pad, it travels through the membrane until it reaches the conjugate pad, where the target microRNA hybridizes with the AuNPs-conjugated capture probe. The sample continues to migrate and is captured by the control probe in the control line. The accumulation of target produces a red band in the test line, the intensity of which is proportional to the amount of target microRNA present in the sample. In the absence of the microRNA, just the control band is observed, ensuring proper liquid flow through the NALFA. MicroRNA detection for POC diagnosis has the disadvantage of requiring microRNA isolation from biological fluids before detection. Emerging technologies for microRNA extraction and detection in portable devices, on the other hand, should increase the current usage of microRNAs for diagnosis (55).

## 6. LFA for the fast detection of MAP infection

Novel LFAs for the fast detection of MAP infection have been summarized in Table 2. A recombinant polymerase amplification-based LFA (RPA-LFA) showed a sensitivity and specificity of 88.24 and 78.75% when compared with a commercial ELISA for the detection of antibodies against MAP (IDEXX, United States) and 100% sensitivity and 97.63% specificity when compared to a real-time qPCR assay (56). The MAP IS9000 sequence was detected in 30 min at 39°C with a limit of detection (LOD) of eight copies per reaction, equivalent to the LOD of the real-time qPCR assay. Subsequently, 612 fecal samples were tested by RPA-LFA, which yielded 100% sensitivity, 97.63% specificity, and 98.44% concordance with the qPCR results.

**Table 2:** LFAs for the detection of MAP infection.

Targeted substrate	Recognition element	Matrix	Performance	Time to detection	Reference
MAP genomic DNA	MAP IS900 fragment	feces	^1^LOD = 8 copies/reaction Sensitivity = 100% Specificity = 97.63%	35 min	Zhao et al. (56)
anti-MAP2963 antibodies	Guinea-pig anti-bovine IgG antibodies	Serum	^2^LOD = 1.98 ul/ml Sensitivity = 84.2% Specificity = 83.3% PPV = 88.89%	7 min	Agrawal et al. (57)
anti-MAP 2677c, 3547c, 4308c, 1693c, and 2168c antibodies	Cocktail of MAP antigens (MAP 2677c, 3547c, 4308c, 1693c, and 2168c)	Serum	^3^Sensitivity = 75.16% Specificity = 100% PPV = 100%	5 min	Jain et al. (58)

(1) RPA-LFA performance was compared with that of the qPCR. (2) and (3) LFAs sensitivities and specificities were compared with that of ELISA. LOD, the limit of detection, PPV, the positive predictive value.

An AuNPs-based LFI for the detection of specific antibodies against MAP2963 protein was developed (57). In this assay, the MAP2963 recombinant protein (44 kDa) and protein A were spotted in the nitrocellulose membrane in the test and control lines, respectively. AuNPs were functionalized with anti-bovine IgG antibodies from guinea pig that can bind to bovine anti-MAP2963 antibodies. MAP2963-specific antibodies in the sample bind to anti-bovine IgG AuNP-conjugated antibodies and, this complex is captured by the 44 KDa recombinant protein, resulting in a red band in the test line. The remaining sample moves further through the nitrocellulose membrane and binds to the protein A, resulting in a red color in the control line. The assay was tested using 31 serum samples collected from cattle with clinical PTB and displayed LOD values of 1.98 μg/mL, sensitivity of 84.2%, specificity of 83.3%, and a positive predictive value (PPV) of 88.89% in comparison to ELISA. There was a good agreement between the ELISA and the LFA assays (kappa value = 0.66). This LFA could be completed in 10 min.

Jain et al. developed a LFI based on a cocktail of MAP 2677c, 3547c, 4308c, 1693c, and 2168c recombinant proteins (each antigen at 2 mg/mL in the cocktail) (58). The MAP antigens were in the test line, whereas biotinylated bovine serum albumin (BSA) was present in the control line. The sensitivity and specificity of the LFI were 75.6 and 100%, respectively. Six hundred and eight animals were tested with this LFI, and 283 were determined to be positive. The kappa value (0.70) revealed a good agreement between the LFI and an ELISA that used the same recombinant secretory proteins as the LFI. In 5 min, LFI results were obtained.

## 7. Applications of LFAs and future directions

Although POC devices for human medicine have made tremendous progress, due primarily to the COVID-19 pandemic, animal POC testing for farm animals has yet to reach its full potential. LFAs could be used to monitor large numbers of animals in the field and to detect antibodies against MAP antigens, as well as bovine microRNAs, proteins or metabolites associated with MAP infection using a relatively small sample volume. Although LFAs for detecting antibodies against MAP antigens have been developed, the next generation of host biomarkers-based LFAs with greater sensitivity for detecting subclinical cases is expected to be developed. Furthermore, multiplex LFAs would enable the simultaneous detection of more than one discriminative biomarker, increasing the detection of the different stages of the infection. In the future, it is expected that mass production techniques would significantly reduce the cost of manufacturing LFAs, making this technology affordable to producers and veterinarians. Besides PTB, LFAs would be especially useful for diagnosing zoonotic mycobacterial infections like bovine tuberculosis which is endemic in low- and middle-income countries with limited access to sophisticated laboratories. Moreover, the development of host biomarker signature based LFAs that are ideally DIVA (allowing vaccination) could be a promising path ahead. The future application of LFAs will require previous evaluation and validation on larger animal cohorts of experimentally and naturally infected cows.

## Author contributions

M-AH: Conceptualization, Formal analysis, Funding acquisition, Methodology, Project administration, Supervision, Writing – original draft, Writing – review & editing. AB: Formal analysis, Investigation, Methodology, Resources, Software, Visualization, Writing – review & editing. AN: Investigation, Methodology, Writing – review & editing. GB-B: Methodology, Software, Visualization, Writing – review & editing. RC: Formal analysis, Funding acquisition, Investigation, Methodology, Project administration, Resources, Supervision, Validation, Writing – original draft, Writing – review & editing.
